# 6-Gingerol attenuates subarachnoid hemorrhage-induced early brain injury via GBP2/PI3K/AKT pathway in the rat model

**DOI:** 10.3389/fphar.2022.882121

**Published:** 2022-08-25

**Authors:** Hui Tang, Chuan Shao, Xiaoya Wang, Yi Cao, Zhou Li, Xiaoquan Luo, Xiang Yang, Yuekang Zhang

**Affiliations:** ^1^ Department of Neurosurgery, Nanchong Central Hospital, The Second Clinical Medical College, North Sichuan Medical College, Nanchong, SC, China; ^2^ Department of Neurosurgery, West China Hospital, Sichuan University, Chengdu, SC, China; ^3^ Department of Neurosurgery, Chongqing General Hospital, University of Chinese Academy of Sciences, Chongqing, China; ^4^ Department of Neurosurgery, Chengdu Second People’s Hospital, Chengdu, SC, China; ^5^ Department of Neurosurgery, The Second Affiliated Hospital of Chongqing Medical University, Chongqing, China

**Keywords:** subarachnoid hemorrhage, 6-gingerol, early brain injury, neuroprotection, GBP2/PI3K/AKT pathway

## Abstract

Numerous studies have elucidated the neuroprotective effect of 6-gingerol in central nervous system diseases. However, the potential role and mechanism of 6-gingerol on early brain injury (EBI) after subarachnoid hemorrhage (SAH) remains poorly understood. Here, we report that 6-gingerol exerts a neuroprotective effect on SAH-induced EBI through the GBP2/PI3K/AKT pathway. A SAH rat model was established by injecting femoral artery blood into the cisterna magna. 6-gingerol or vehicle was injected intraperitoneally 1 hour post-SAH induction. We found that the neurological function score and brain edema of SAH rats were significantly improved after 6-gingerol treatment, as well as neuronal apoptosis was attenuated in SAH rats by Nissl staining assay and TUNEL assay. To further explore potential molecular mechanisms associated with 6-gingerol, RNA sequencing was implemented to investigate the differences in transcriptomes between SAH rats with and without 6-gingerol treatment; and found that the expression of guanylate-binding protein 2 (GBP2) evidently was suppressed with 6-gingerol treatment compared to vehicle group. In addition, dual immunofluorescence was also employed to investigate changes in neurons, astrocytes, and microglia after 6-gingerol treatment. The results showed that GBP2 was expressed in neurons but not astrocytes or microglia. Western blotting analysis results demonstrated that the PI3K/AKT pathway was activated in the SAH rats treated with 6-gingerol. Furthermore, recombinant GBP2 protein and LY294002 (PI3K inhibitor) treatment reversed the effects of 6-gingerol treatment in SAH rats. These results indicate that 6-gingerol suppressed the expression of GBP2 to activate the PI3K/AKT pathway, improve neurologic outcomes, reduce brain edema and neuronal apoptosis. In summary, our findings suggest that 6-gingerol could attenuate EBI post-SAH in rats, and 6-gingerol may serve as a novel candidate neuroprotective drug for SAH-induced EBI.

## Introduction

Subarachnoid hemorrhage (SAH) is a common cerebrovascular disease that exhibits high morbidity and mortality rates (Chen et al., 2020), and mainly caused by the rupture of intracranial aneurysms. With the development of medical imaging, microsurgical and neurointerventional techniques, the mortality caused by a ruptured intracranial aneurysmal rebleed has been moderately reduced (Daou et al., 2019). However, blood components and secondary degradation products in the subarachnoid space cannot be removed quickly and effectively and evoke brain injury post-SAH. Thus, mortality and disability remain very high in SAH, which imposes a heavy burden on families and society (Suwatcharangkoon et al., 2016).

Early brain injury (EBI), which encompasses the entirety of brain injury occurring in the 72 h post-SAH, contributes to poor outcomes (Rass and Helbok, 2019; Neifert et al., 2021). Multiple factors at early stages post-SAH, including increased intracranial pressure, decreased cerebral perfusion, global ischemia, oxidative stress, microcirculatory failure and neuroinflammation, ultimately result in apoptotic neuronal cell death (van Lieshout et al., 2018; Rass and Helbok, 2019). Furthermore, it has been shown that excessive neuronal apoptosis leads to an unfavorable neurological prognosis. Therefore, inhibition of neuronal apoptosis is a crucial therapeutic strategy for neurological functional recovery post-SAH. Currently, however, effective drug treatment for SAH is still very limited.

6-gingerol is a natural compound extracted from ginger and has been reported to exhibit potent effects against oxidation, inflammation, apoptosis and cancer (Chen et al., 2018; de Lima et al., 2018; Zhang et al., 2018; Luo J. et al., 2021; Rezazadeh-Shojaee et al., 2021). Previous studies have confirmed that 6-gingerol ameliorates apoptosis under different experimental conditions in various cells and organs, such as hypoxia-induced cardiomyocytes (Zhang et al., 2019), myocardial ischemia/reperfusion injury (Lv et al., 2021), myocardial fibrosis (Han et al., 2020), osteoarthritis (Abusarah et al., 2017), renal damage (Hegazy et al., 2016) and atherosclerosis (Yang et al., 2020). In addition, 6-gingerol has excellent blood-brain barrier permeability, resulting in direct effects on brain tissue (Simon et al., 2020). Importantly, relevant studies have confirmed that 6-gingerol also exhibits neuroprotective effects against hypoxic-ischemic brain injury (Zhao et al., 2021), cerebral ischemia/reperfusion injury (Luo Y. et al., 2021), amnesia (Kim et al., 2018), acrylonitrile-induced brain damage (Farombi et al., 2018) and Alzheimer’s disease (Rezazadeh-Shojaee et al., 2021). However, the protective effects of 6-gingerol against EBI post-SAH remain unclear.

In this study, we investigated the neuroprotective effects and underlying mechanism employed by 6-gingerol against EBI post-SAH, and found that 6-gingerol inhibited neuronal apoptosis to improve neurological deficits in a SAH-induced EBI rat model. Moreover, we found that 6-gingerol activates the PI3K/AKT pathway via guanylate-binding protein 2 (GBP2) inhibition to suppress neuronal apoptosis in SAH rats. The current findings will provide a theoretical basis for potential therapeutic drugs, leading to new therapies for patients with post-SAH EBI.

## Materials and methods

### Animals

All animal experimental protocols were approved by the Ethics Committee of North Sichuan Medical College (Protocol No. 2021–57, Nanchong, China). Adult male Sprague-Dawley rats (250 ± 10 g) were purchased from Charles River Laboratories (Beijing, China) and housed in the Experimental Animal Center of North Sichuan Medical College (Nanchong, China). The rats were raised in a controlled environment (temperature: 23 ± 2°C, light/dark cycle: 7:30 a.m./7:30 p.m.) with free access to food and water.

### Experimental SAH model

The experimental SAH model was produced as previously described (Zhao et al., 2019). Briefly, after intraperitoneal anesthesia with pentobarbital (35 mg/kg), a syringe was inserted into the femoral artery under sterile procedures to withdraw blood. Then, the rats were rotated to the prone position and placed in a stereotactic frame. Approximately 0.20 ml blood was then slowly injected into the cisterna magna for 2 min under aseptic conditions, whereas 0.20 ml saline was used for the sham group. After injection, the rats were kept at a constant temperature of 30°C, in a heads-down position for 30 min and injected subcutaneously with 5 ml saline to prevent dehydration. After the operation, the rats were returned to their cages.

### Experimental design and drug administration

This experiment was divided into five parts (Supplementary Figure S1).

In experiment 1, to identify the role of 6-gingerol on SAH rat, 72 rats (a total of 64 rats were used, of which eight died after induction of SAH) were assigned randomly into four groups: sham, SAH + phosphate buffered saline (PBS), SAH + 6-gingerol (5 mg/kg) and SAH + 6-gingerol (10 mg/kg). 6-gingerol (Yuanye BioTechnology, Shanghai, China) was dissolved in PBS and injected intraperitoneally 1 h post-SAH induction. Neurological score (n = 6) and brain water content (n = 6) were determined and Nissl staining (n = 5), terminal deoxynucleotidyl transferase dUTP nick end labeling (TUNEL) staining (n = 6) and western blotting (n = 5) were carried out at 24 h post-SAH.

In experiment 2, to investigate the mechanisms of 6-gingerol under SAH conditions, eight rats (six rats were used, two rats died post-SAH) were assigned randomly into two groups: SAH + PBS and SAH + 6-gingerol (10 mg/kg) (n = 3/group) and RNA-sequencing was performed at 24 h post-SAH.

In experiment 3, to determine the expression pattern and distribution of GBP2 post-SAH, rats were randomly divided into sham group and SAH + PBS groups with different time points (6, 12, 24, 48 and 72 h). The brain tissues from each group were collected for western blot (n = 5). In addition, 40 rats were assigned randomly into four groups: sham, SAH + PBS, SAH + 6-gingerol (5 mg/kg) and SAH + 6-gingerol (10 mg/kg) group. Western blotting (n = 10, n = 5 in SAH + 6-gingerol (5 mg/kg) group) was conducted to detect GBP2 expression levels and immunofluorescence assays were performed to determine the specific location of GBP2 expression at 24 h post-SAH.

In experiment 4, to evaluate the relation between 6-gingerol and GBP2 in SAH rats, 1 μg rGBP2 (1μg/10 μL PBS) recombinant GBP2 (rGBP2) protein was injected intracerebroventricularly using a 25 μL micro-injection needle (Gaoge, Shanghai, China) 2 h post-SAH induction under anesthesia (coordinates: 1.5 mm behind the bregma and 1.0 mm lateral from the sagittal midline, 4 mm depth from the skull surface), the needle was left for at least 5 min and the burr hole was plugged immediately with bone wax to prevent the leakage from the injection after the administration, and 89 rats (80 rats were used, nine rats died post-SAH) were then randomly divided into sham, SAH + PBS, SAH + 6-gingerol (10 mg/kg) and SAH + 6-gingerol (10 mg/kg) + rGBP2 groups. Neurological score (n = 6) and brain water content (n = 6) were assessed, and western blotting (n = 5) and Nissl (n = 5) and TUNEL staining (n = 5) were performed at 24 h post-SAH.

In experiment 5, to identify whether 6-gingerol affected the PI3K/AKT pathway, LY294002, a selective inhibitor of PI3K signaling was introduced, and 64 rats (55 rats were used, nine rats died post-SAH) were randomly divided into sham, SAH + PBS group, SAH + 6-gingerol (10 mg/kg), SAH + 6-gingerol (10 mg/kg) + LY294002 and SAH + 6-gingerol (10 mg/kg) + DMSO groups (n = 11/group). Ten μL of LY294002 solution (50 mmol/L in 25% DMSO in PBS) was injected intracerebroventricularly (as mentioned in experiment 4) 30 min prior to SAH induction. Neurological score (n = 6) and brain water content (n = 6) were assessed, and western blotting (n = 5) was performed at 24 h post-SAH.

### Neurological score

The neurological performances of rats were evaluated at 24 h post-SAH by a blinded investigator using a 6-point scoring system (Wang et al., 2012). Three behavioral activity examinations, including appetite, activity and neurological deficits were used in the scoring methodology. Details on the scoring procedures are shown in Table 1.

**TABLE 1:** Neurobehavioral evaluation.

Category	Behavior	Score
Appetite	Finished meal	0
	Left meal unfinished	1
	Scarcely ate	2
Activity	Walk and reach at least three corners of the cage	0
	Walk with some stimulations	1
	Almost always lying down	2
Deficits	No deficits	0
	Unstable walk	1
	Impossible to walk	2

### Brain water content

At 24 h post-SAH, rats were anesthetized, and whole brain tissues were removed. Blood from the brain surface was gently blotted with filter paper, and the whole brains were immediately weighed (recorded as the wet weight). Then, the brains were dried for 72 h at 100°C and weighed again to obtain the dry weight. The percentage of brain water content was calculated as [(wet weight − dry weight)/wet weight] × 100%.

### TUNEL staining

TUNEL staining was carried out using the Cell Death Detection Kit (Roche, San Francisco, CA, United States) according to the manufacturer’s protocol. Briefly, the basal temporal lobes were paraffin-embedded and sectioned (5 μm). Subsequently, brain slides were deparaffinized, dehydrated and incubated with the TUNEL reaction mixture for 1 h at 37°C. Nuclei were stained with 4′,6-diamidino-2-phenylindole (DAPI) (Solarbio, Beijing, China) mounting medium after being washed three times with PBS at room temperature. Finally, the sections were observed by a fluorescence microscope (Leica, Oberkochen, Germany) and TUNEL-positive cells were counted by a researcher who was blind to the experimental groups.

### Nissl staining

Nissl staining was applied to measure neuronal loss in brain tissues as previously described. In brief, after being deparaffinized and hydrated, the basal temporal lobe sections were incubated with methyl violet staining solution for 20 min at 37°C and used Nissl differentiation to differentiate for 4–8 s, and were then dehydrated with graded alcohols and cleared in xylene. Images of stained brain sections were captured and analyzed under a light microscope (Leica) by a researcher who was blinded to the experimental conditions.

### Western blotting analysis

The basal temporal brain tissue was washed thoroughly with pre-cooled PBS to remove the blood. Weigh, smash and homogenate the tissue, total protein was extracted using protein lysis buffer (Beyotime, Shanghai, China) according to the ratio of 100 mg tissue to 1 ml buffer. Protein concentration was determined with a BCA kit (Solarbio, Beijing, China). The same amounts of total protein (30 ug) were separated by electrophoresis in 10–12% sodium dodecyl sulfonate-polyacrylamide gel and transferred to polyvinylidene difluoride membranes (Millipore, Billerica, MA, United States). Then, the membranes were blocked with 5% nonfat dry milk/TBST for 1 h at room temperature before being incubated with specific primary antibodies overnight at 4°C. The membranes were incubated with horseradish peroxidase-linked secondary antibodies for 1 h at room temperature. Finally, signals were detected by SuperSignal ECL (Millipore, Billerica, MA, United States). The relative quantity of proteins was analyzed by using ImageJ software (National Institutes of Health, Rockville, United States). The primary antibodies were as follows: anti-GBP2 (1:200, sc-271568, Santa Cruz), anti-bax (1:1,000, ab32503, Abcam), anti-bcl-2 (1:1,000, ab194583, Abcam), anti-caspase 8 (1:1,000, ab25901, Abcam), anti-PI3K (1:1,000, 4,292, CST), anti-p-PI3K (1:1,000, 4,228, CST), anti-AKT (1:1,000, 9,272, CST), anti-p-AKT (1:1,000, 4,060, CST) and anti-β-actin (1:5,000, AC026, ABclonal).

### RNA sequence

Total RNA was extracted from the designated detection site in the basal temporal lobes of rats either treated with 6-Gingerol (10 mg/kg) or control PBS using TRIZOL reagent (Invitrogen). RNA extraction, library preparation, RNA-seq, and bioinformatics analysis were performed at CapitalBio Technology Co., Ltd., (Beijing, China). Briefly, 2 μg of total RNA was isolated using the Qiagen RNeasy Kit (Qiagen, Valencia, CA, United States), and libraries were sequenced on the Illumina HiSeq2000 system (Illumina, San Diego, CA, United States). Sequence reads are analyzed with the STAR alignment-DESeq2 software pipeline described in the Data Explanation document. The list of differentially expressed genes from DESeq2 output was selected based on 10% adjusted *p*-value level and false discovery rate value of 0.10. Gene Ontology and KEGG pathway enrichment analysis were done using the DAVID bioinformatics resources portal. Three replicates for each group were sequenced. Differential expressed genes with log2 (fold change) > 1 and *p* < 0.05 were considered as significant.

### Immunofluorescence assay

Rats of each group were anesthetized and underwent perfusion with cold normal saline followed by 4% cold paraformaldehyde (PFA) to fix the tissue. Brains were removed and postfixed in 4% PFA for 24 h, dehydrated in 30% sucrose for 72 h and then cut the basal temporal lobes into serial 15 μm thick coronal sections using a cryo microtome (Leica). For immunofluorescence staining, the sections were incubated overnight at 4°C with the following primary antibodies: rabbit anti-NeuN (1:200; Abcam, United States), rabbit anti-GFAP (1:200; Abcam, United States), rabbit anti-Iba-1 (1:100; Abcam, United States) and mouse anti-GBP2 (1:50; Santa Cruz, United States). Next, the sections were incubated with fluorescence conjugated secondary antibodies for 1 h at room temperature before DAPI staining (zsbio, China). Fluorescence microscopy was adopted to observe and capture images (Leica).

### Statistical analysis

GraphPad Prism 8.0 (GraphPad Software, San Diego, CA, United States) was employed for statistical analysis. The neurological score was presented as the median ± the interquartile range, and the Mann-Whitney *U* test was used to compare differences among groups. Other data were presented as the mean ± standard deviation, the one-way ANOVA test was used to compare differences between multiple groups, and the Tukey post hoc test was used for intergroup comparisons. Statistical significance was accepted at *p* < 0.05.

## Results

### 6-Gingerol significantly improves neurologic function and reduces brain edema post-SAH

**FIGURE 1:**
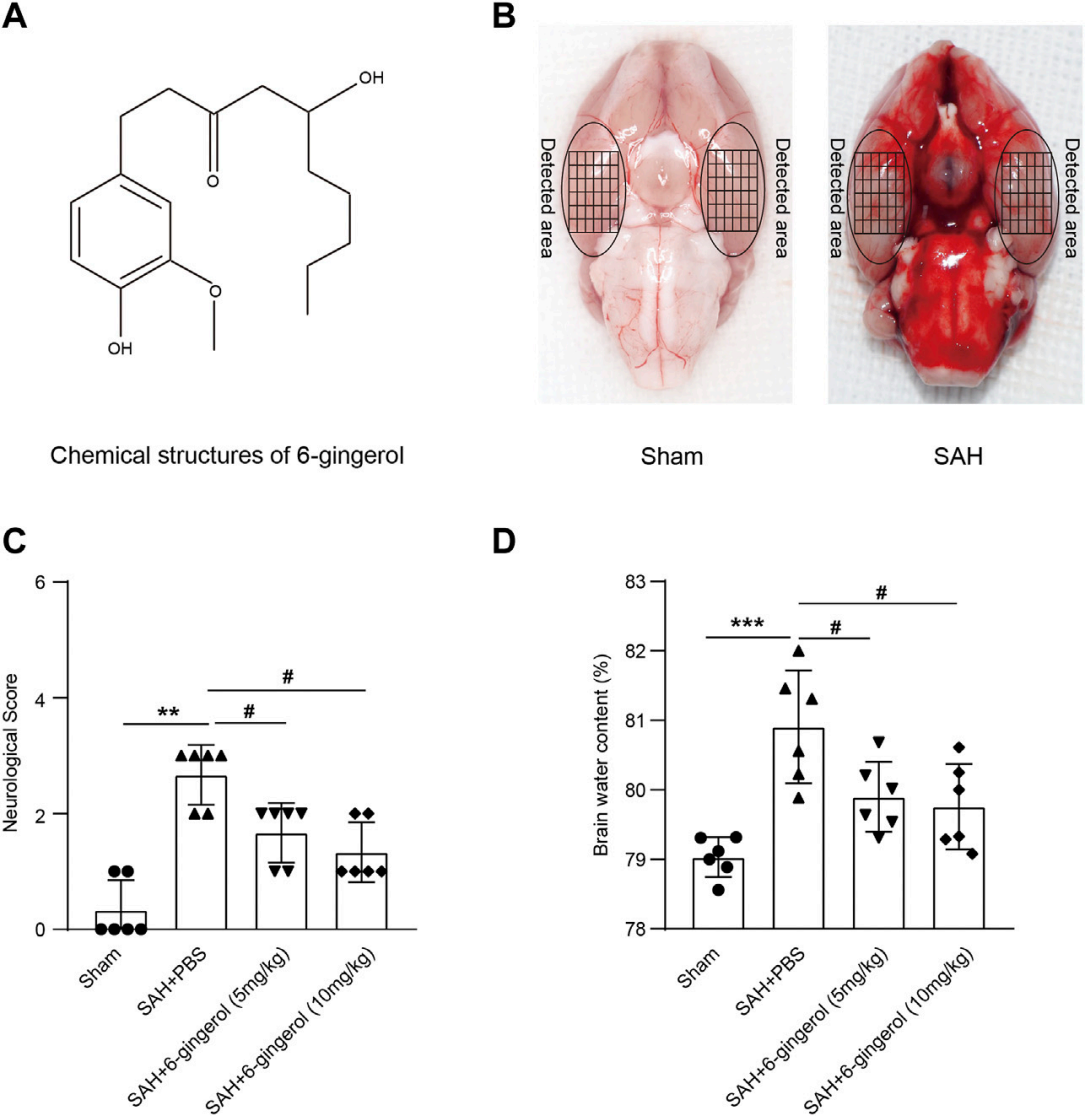
6-gingerol improves neurologic function and reduces brain edema in SAH rats. **(A)** The chemical structure of 6-gingerol. **(B)** Representative images of the SAH rat brain and the sham rat brain. **(C)** Quantitative analysis of neurological scores, n = 6, Mann-Whitney *U* test was used. **(D)** Quantitative analysis of brain water content, n = 6, One-way ANOVA was used followed by Tukey’s post hoc test. ^**^ *p* < 0.01, ^***^ *p* < 0.001 vs sham group; ^#^ *p* < 0.05 vs SAH + PBS group.

The chemical structure of 6-gingerol and representative brain images of sham and SAH rats are shown in Figure 1A and Figure 1B, respectively. The Mortality and excluded rat numbers per group are presented in Supplementary Table S1. To identify the effects of 6-gingerol on the neurological deficit and brain edema post-SAH, we assessed neurological scores in accordance with Table 1 and detected the brain water content, respectively, in rats at 24 h post-SAH. The results revealed that the neurological scores of 6-gingerol treatment SAH group significantly decreased compared with the SAH + PBS group (*p* < 0.05, Figure 1C). Moreover, brain edema test results showed that the brain water content of the SAH + 6-gingerol group clearly decreased compared with the SAH + PBS group (*p* < 0.05, Figure 1D). Brain edema and neurological deficit are mainly responsible for poor outcomes in post-SAH EBI. Thus, our results indicate that 6-gingerol may exert a protective effect in post-SAH EBI.

### 6-Gingerol significantly suppresses neuronal apoptosis post-SAH

**FIGURE 2:**
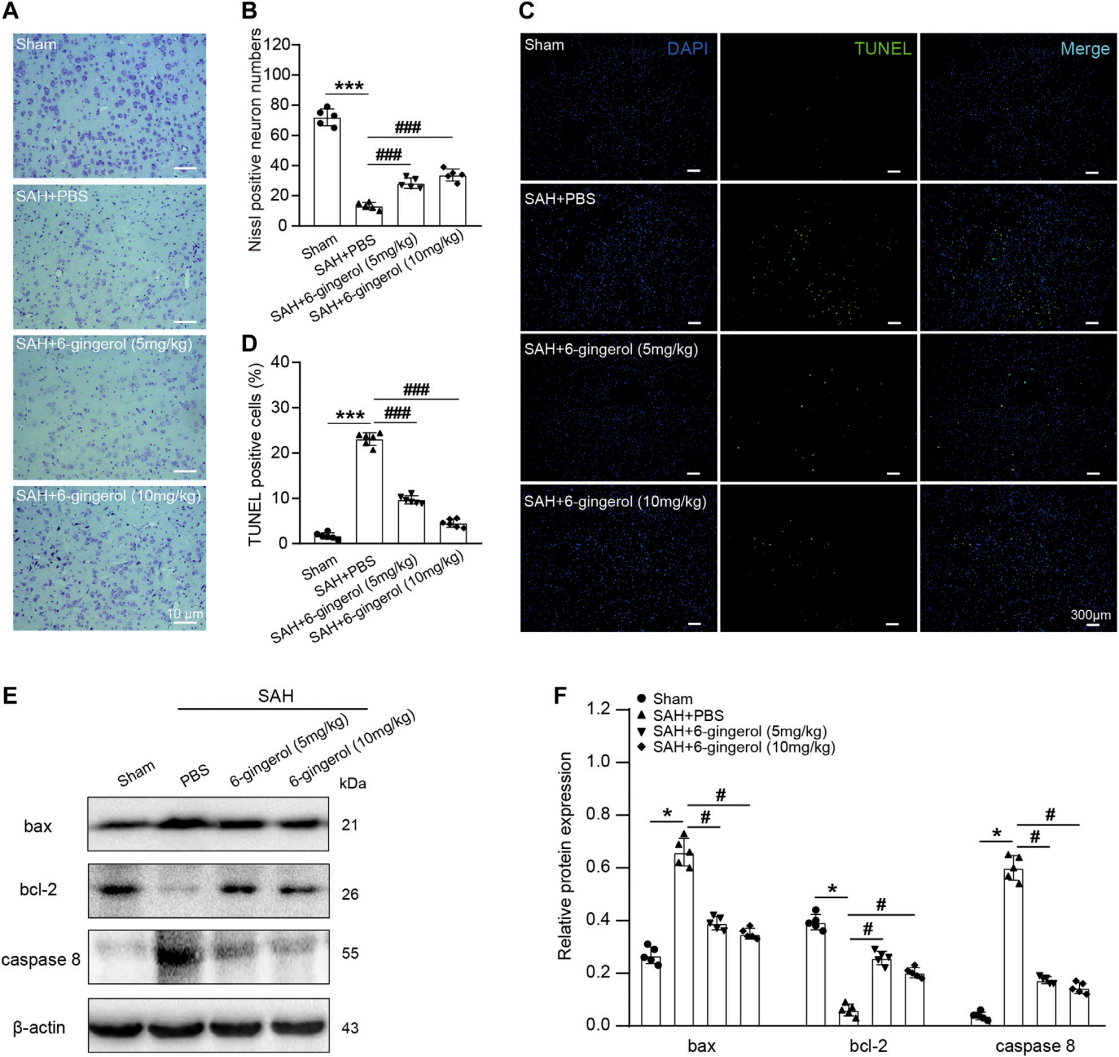
6-gingerol alleviates neuronal apoptosis in SAH rats. **(A)** Representative images of Nissl staining. Scale bar = 10 μm. **(B)** Quantitative analysis of Nissl-positive neuron numbers, n = 5. One-way ANOVA was used followed by Tukey’s post hoc test. **(C)** Representative images of TUNEL staining. Scale bar = 300 μm. **(D)** Quantitative analysis of TUNEL-positive cells, n = 6. One-way ANOVA was used followed by Tukey’s post hoc test. **(E)** Bax, bcl-2 and caspase 8 protein expression were detected by western blot, n = 5. **(F)** Quantitative western blot. One-way ANOVA was used followed by Tukey’s post hoc test. ^*^ *p* < 0.05, ^***^ *p* < 0.001 vs sham group; ^#^ *p* < 0.05, ^###^ *p* < 0.001 vs SAH + PBS group.

We next determined the effects of 6-gingerol on neuronal apoptosis. Nissl staining revealed that the SAH + PBS group had fewer viable neurons than the sham group (*p* < 0.001), while an increased number of viable neurons was observed in the SAH + 6-gingerol group (*p* < 0.001 for each, Figures 2A,B). Additionally, TUNEL staining results demonstrated that rats subject to SAH exerted more frequent cell apoptosis compared with the sham group (*p* < 0.001), while 6-gingerol administration dramatically brought down the apoptotic ratio in rat brain post-SAH (*p* < 0.001 for each, Figures 2C,D). Furthermore, we conducted western blotting analysis to evaluate apoptosis markers. Compared with the SAH + PBS group, caspase 8 and bax protein expression was significantly reduced in the SAH + 6-gingerol group, while bcl-2 protein expression was increased in the SAH + 6-gingerol group (*p* < 0.05 for each, Figures 2E,F). Collectively, these data indicate that 6-gingerol treatment has a significant anti-apoptotic effect on neuronal cells of rats post-SAH.

### 6-Gingerol inhibits GBP2 expression post-SAH

**FIGURE 3:**
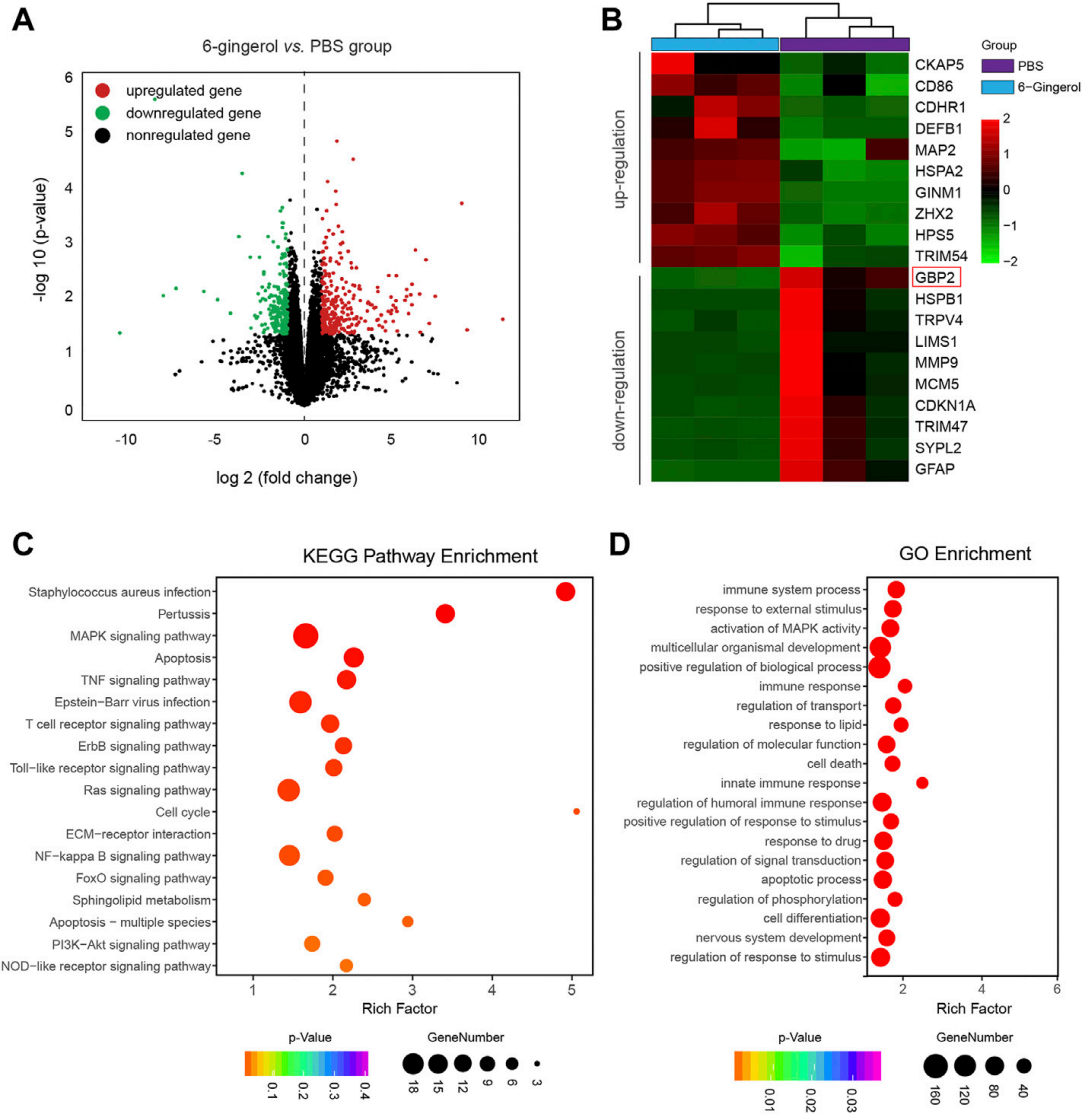
The transcriptomes of SAH + 6-gingerol rats and SAH + PBS rats. **(A)** The volcano map displays differentially expressed genes in SAH rats with or without 6-gingerol treatment (*p* < 0.05, log(fold change) > 1). Red and green represent upregulated and downregulated genes, respectively, and black represents nonregulated genes. **(B)** The heatmap shows the list of genes with the most significant differences between SAH + 6-gingerol rats and SAH + PBS rats. The color key from green to red represents the log2 from low to high expression. **(C)** KEGG enrichment analysis. The *y*-axis and *x*-axis indicate pathway name and rich factor, respectively. The sizes and colors of bubbles represent the gene number and enrichment *p*-value in the corresponding pathway, respectively. **(D)** GO enrichment analysis. The sizes and colors of bubbles indicate the enriched genes number and adjusted *p*-value, respectively.

To further explore the molecular mechanism of 6-gingerol in SAH rat, we performed an RNA-sequencing approach to compare the transcriptomes of rats in the SAH + 6-gingerol group and those in the SAH + PBS group. As shown in Figure 3A, 566 genes were differentially expressed at levels greater than two-fold following 6-gingerol treatment, including 305 genes up-regulated and 261 down-regulated genes. Among the down-regulated genes, *GBP2* was expressed at the lowest levels in rats of the SAH + 6-gingerol group (Figure 3B).

**FIGURE 4:**
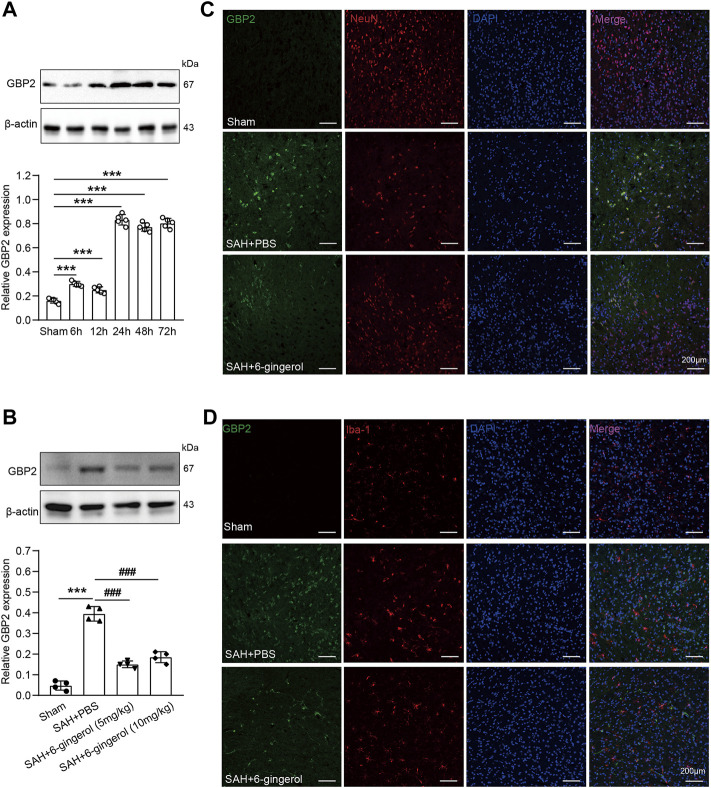
6-gingerol inhibits GBP2 expression in SAH rats. **(A)** GBP2 protein expression was determined by immunoblot at preset times post-SAH and quantitative western blot, n = 5. One-way ANOVA was used followed by Tukey’s post hoc test. **(B)** GBP2 protein expression was evaluated by western blotting in sham, SAH + PBS, SAH + 6-gingerol (5 mg/kg) and SAH + 6-gingerol (10 mg/kg) rats, and quantitative western blot, n = 4. One-way ANOVA was used followed by Tukey’s post hoc test. **(C)** Representative images of GBP2/NeuN immunofluorescence staining of brain sections from rats in the sham, SAH + PBS and SAH + 6-gingerol groups. Scale bar = 200 μm. **(D)** Representative images of GBP2/Iba-1 immunofluorescence staining of brain sections from rats in the sham, SAH + PBS and SAH + 6-gingerol groups. Scale bar = 200 μm ^***^ *p* < 0.001 vs sham group; ^###^ *p* < 0.001 vs SAH + PBS group.

Next, the genes whose expression was down-regulated more than two-fold were selected to analyze pathway enrichment to identify the function of 6-gingerol in SAH rats. Kyoto Encyclopedia of Genes and Genomes (KEGG) and gene ontology (GO) enrichment analyses results showed that cell apoptosis and immune response had a high correlation 6-gingerol treatment (Figures 3C,D). Then, we performed immunoblot analysis to detect the GBP2 expression in SAH rats, and the results revealed that GBP2 protein expression significantly increased and reached a peak at 24 h post-SAH (*p* < 0.05 for each, Figure 4A). Moreover, western blotting showed that GBP2 expression was down-regulated in SAH rats treated with 6-gingerol (*p* < 0.001, Figure 4B). The immunofluorescence staining showed that neuron number was reduced in SAH + PBS group compared with the sham group rat, while the neuron number in SAH + 6-gingerol group rats exceeded compared with the SAH + PBS group; the number of microglia increased in SAH + PBS group rats compared with sham group, and number of microglia in SAH + 6-gingerol group rats was less than SAH + PBS group. However, the number of astrocytes was not different among the groups (Supplementary Figure S2). To investigate the role of GBP2 in neurons and microglia, double immunofluorescence staining for GBP2 and NeuN and GBP2 and Iba-1, respectively, were performed. The data revealed that GBP2 expression significantly increased in SAH rats compared with controls, while it obviously decreased 6-gingerol treated rats compared with SAH rats. In addition, we found extensive GBP2/NeuN co-localization (Figure 4C). Unexpectedly, we observed little GBP2/Iba-1 co-localization (Figure 4D). Collectively, these data indicate that 6-gingerol can inhibit GBP2 expression post-SAH, and that GBP2 may directly mediate the function of neurons.

### 6-Gingerol inhibits GBP2-mediated neuronal apoptosis post-SAH

**FIGURE 5:**
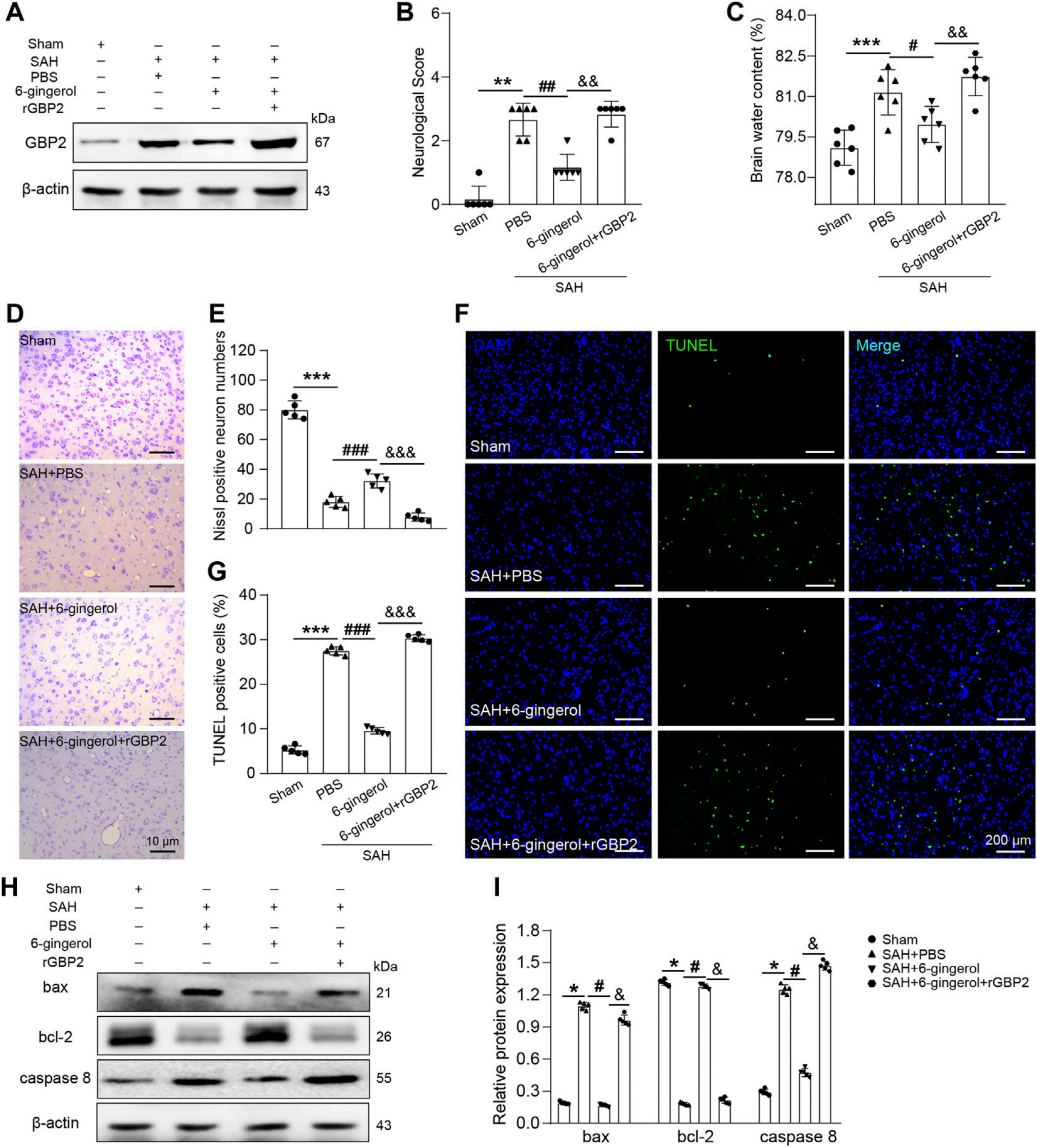
6-gingerol inhibits GBP2-mediated neuronal apoptosis in SAH rats. **(A)** GBP2 protein expression was detected by western blotting in rats of the sham, SAH + PBS, SAH + 6-gingerol and SAH + 6-gingerol + rGBP2 groups. **(B)** Quantitative analysis of neurological scores, n = 6, Mann-Whitney *U* test was used. **(C)** Quantitative analysis of brain water content, n = 6. One-way ANOVA was used followed by Tukey’s post hoc test. **(D)** Representative images of Nissl staining. Scale bar = 10 μm. **(E)** Quantitative analysis of Nissl-positive neuron numbers, n = 5. One-way ANOVA was used followed by Tukey’s post hoc test. **(F)** Representative images of TUNEL staining. Scale bar = 200 μm. **(G)** Quantitative analysis of TUNEL-positive cells, n = 5. One-way ANOVA was used followed by Tukey’s post hoc test. **(H)** Bax, bcl-2 and caspase 8 protein expression were evaluated by western blotting. **(I)** Quantitative western blot, n = 5. One-way ANOVA was used followed by Tukey’s post hoc test. ^*^ *p* < 0.05, ^**^ *p* < 0.01, ^***^ *p* < 0.001 vs sham group; ^#^ *p* < 0.05, ^##^ *p* < 0.01, ^###^ *p* < 0.001 vs SAH + PBS group; ^&^ *p* < 0.05, ^and&^ *p* < 0.01, ^andand&^ *p* < 0.001 vs SAH + 6-gingerol group.

To investigate whether GBP2 mediates neuronal apoptosis in SAH rats treated with 6-gingerol, rGBP2 was intracerebroventricularly injected 2 h post-SAH. Western blotting analysis showed that GBP2 protein expression significantly increased in the SAH + 6-gingerol + rGBP2 group compared with the SAH + 6-gingerol group (Figure 5A). Next, neurological scores and brain water content detection were performed, and the data revealed that the scores and brain water content of the SAH + 6-gingerol + rGBP2 group significantly increased compared with the SAH + 6-gingerol group (*p* < 0.01 for each, Figures 5B,C). Furthermore, Nissl and TUNEL staining results showed that the total number of neurons and apoptotic cells significantly decreased and increased in SAH + 6-gingerol + rGBP2 rats compared with SAH + 6-gingerol rats, respectively (*p* < 0.001 for each, Figures 5D–G). Immunoblot analysis demonstrated that bax and caspase 8 protein expression were significantly up-regulated, while bcl-2 was down-regulated in the SAH + 6-gingerol + rGBP2 group (*p* < 0.05 for each, Figures 5H,I). Taken together, these data suggest that 6-gingerol inhibits GBP2-mediated neuronal apoptosis to improve neurologic function and reduce brain edema post-SAH.

### 6-Gingerol inhibits GBP2-mediated neuronal apoptosis by activating the PI3K/AKT signaling post-SAH

**FIGURE 6:**
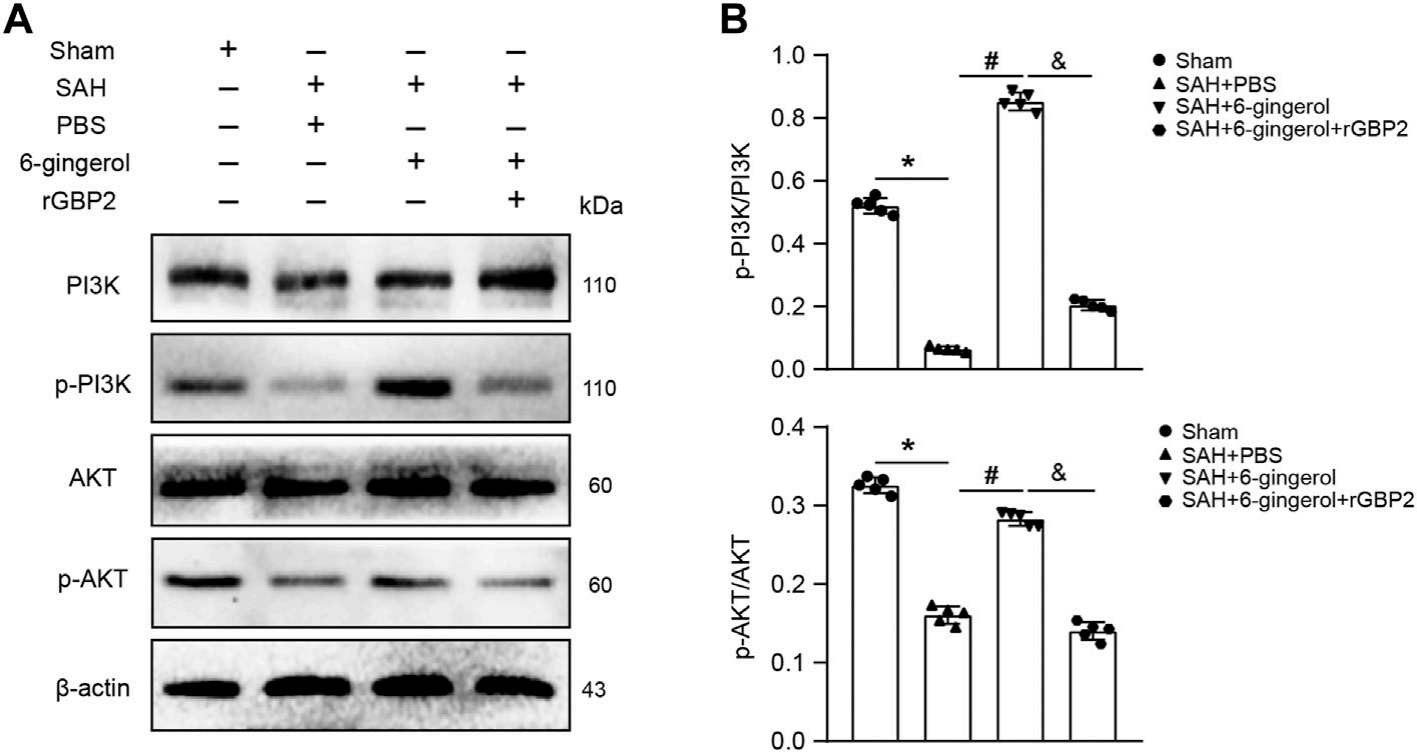
6-gingerol activates the PI3K/AKT pathway to inhibit GBP2-mediated neuronal apoptosis in SAH rats. **(A)** Western blotting analysis of PI3K, p-PI3K, AKT, p-AKT, bax, bcl-2 and caspase 8 protein expression. **(B)** Quantitative western blot, n = 5. One-way ANOVA was used followed by Tukey’s post hoc test. ^*^ *p* < 0.05 vs sham group; ^#^ *p* < 0.05 vs SAH + PBS group; ^&^ *p* < 0.05 vs SAH + 6-gingerol group.

To understand the downstream molecular mechanisms of GBP2, we further analyzed transcriptome sequencing data and found the PI3K/AKT pathway had a high functional enrichment score. Western blotting was performed to detect PI3K/AKT signaling in rats of the sham, SAH + PBS, SAH + 6-gingerol and SAH + 6-gingerol + rGBP2 groups. The data showed PI3K/AKT signaling was reduced in SAH + PBS group compared with the sham group (*p* < 0.05 for each), but was increased in the SAH + 6-gingerol group (*p* < 0.05 for each). Furthermore, p-PI3K and p-AKT protein expression was significantly reduced when in the SAH + 6-gingerol + rGBP2 group compared with the SAH + 6-gingerol group (*p* < 0.05 for each, Figures 6A,B). These results indicate that 6-gingerol inhibits GBP2-mediated neuronal apoptosis by activating PI3K/AKT signaling.

### 6-Gingerol improves neurologic function and reduces brain edema via a GBP2/PI3K/AKT signaling axis post-SAH.

**FIGURE 7:**
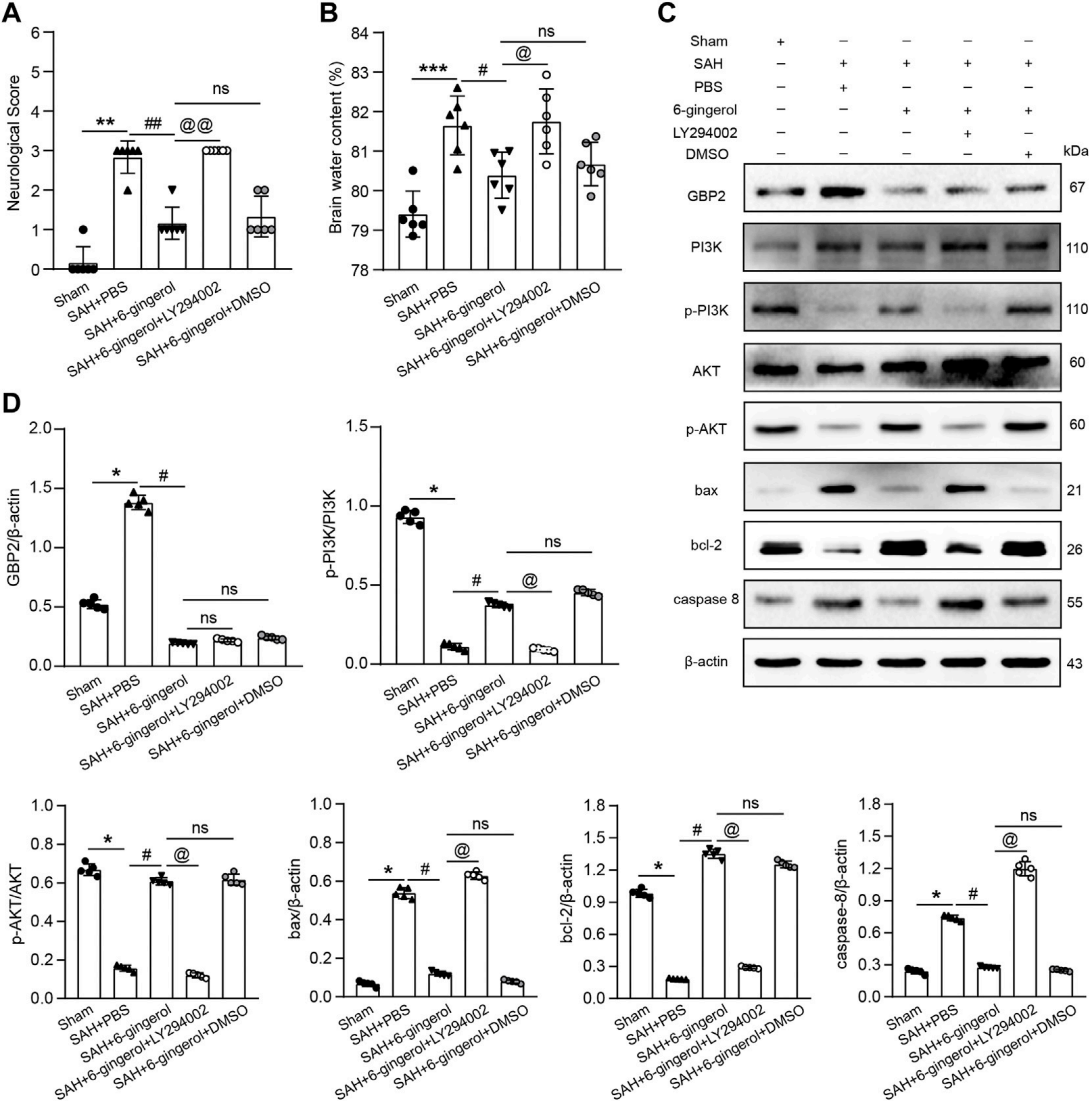
6-gingerol improves neurologic function and reduces brain edema via the GBP2/PI3K/AKT axis in SAH rats. **(A)** Quantitative analysis of neurological scores, n = 6, Mann-Whitney *U* test was used. **(B)** Quantitative analysis of brain water content, n = 6. One-way ANOVA was used followed by Tukey’s post hoc test. **(C)** Immunoblot analysis of GBP2, PI3K, p-PI3K, AKT, p-AKT, bax, bcl-2 and caspase 8 protein expression. **(D)** Quantitative western blot, n = 5. One-way ANOVA was used followed by Tukey’s post hoc test. ^*^ *p* < 0.05, ^**^ *p* < 0.01, ^***^ *p* < 0.001 vs sham group; ^#^ *p* < 0.05, ^##^ *p* < 0.01 vs SAH + PBS group; ^@^ *p* < 0.05, ^@^ *p* < 0.01 vs SAH + 6-gingerol group; ^ns^ *p* = no significant vs SAH + 6-gingerol group.

To investigate whether PI3K/AKT signaling is involved in neurologic function post-SAH, we intracerebroventricularly injected LY294002 (a selective inhibitor of PI3K signaling) 30 min prior to SAH induction to inhibit PI3K/AKT signaling. Neurological scores and brain water content detection were performed, and the data revealed that the scores and brain water content of the SAH + 6-gingerol + LY294002 group significantly increased compared with the SAH + 6-gingerol group (*p* < 0.01, *p* < 0.001, respectively, Figures 7A,B). Western blotting analysis revealed that p-PI3K, p-AKT and bcl-2 were down-regulated in the SAH + 6-gingerol + LY294002 group compared with the SAH + 6-gingerol or SAH + 6-gingerol + DMSO groups, while bax and caspase 8 were up-regulated (*p* < 0.05 for each, Figures 7C,D). Collectively, these data indicate that 6-gingerol improves neurologic function and reduces brain edema via a GBP2/PI3K/AKT signaling axis post-SAH.

## Discussion

**FIGURE 8:**
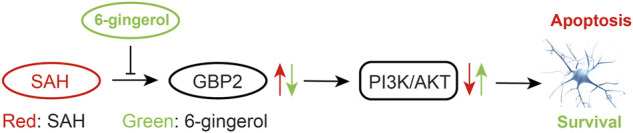
A Schematic representation of signaling pathways showing that 6-gingerol alleviates neuronal apoptosis. GBP2 was up-regulated in SAH, which may promote SAH-induced neuronal apoptosis via inhibition of the PI3K/AKT pathway. However, 6-gingerol can inhibit GBP2-mediated neuronal apoptosis by reactivating the PI3K/AKT signaling post-SAH.

The present study found that 6-gingerol exerted a neuroprotective effect on SAH-induced EBI in rats. Specifically, 6-gingerol improved neurological deficits in SAH rats, alleviating brain edema and reducing neuronal apoptosis. Furthermore, we identified that 6-gingerol suppresses SAH-induced neuronal apoptosis by inhibiting GBP2 expression to upregulate the PI3K/AKT pathway, as Figure 8 shows. Taken together, 6-gingerol may act as a candidate preventive or protective drug for SAH-induced EBI.

EBI is recognized by most scholars as a critical pathological process of SAH (Macdonald, 2014). Over the past decade, many researchers have focused on the mechanism of EBI and concluded that vasospasm, apoptosis, necrosis, inflammatory, oxidative stress, calcium overload and metabolism of neurons might derive the development of EBI (Fujii et al., 2013). Based on these mechanisms, a wide variety of drugs have been used in research and therapy for EBI post-SAH. However, the results of multicenter randomized control trials have indicated that most drugs are ineffective or inefficient for treating EBI post-SAH (Macdonald et al., 2011; Chen et al., 2020). Therefore, more effective and well-defined drugs are urgently needed to treat EBI post-SAH. Early neuronal apoptosis is the primary and most direct mode of neuronal death post-SAH (Croci et al., 2021) and our study aimed to find molecules that inhibit neuronal apoptosis to improve EBI outcomes.

Natural plant drugs have been frequently used for disease treatment, and in current clinical trials, more than 50% of drugs are synthesized from natural compounds (Sekiwa et al., 2000). Among them, 6-gingerol, which is a major active phenolic compound extracted from ginger, attracted our interest. Previous studies demonstrated 6-gingerol to be a potent anti-tumor agent in a variety kind of tumors, including breast cancer (Luna-Dulcey et al., 2021), gastric cancer (Mansingh et al., 2018), lung cancer (Tsai et al., 2020) and renal cell carcinoma (Xu et al., 2020). Besides, 6-gingerol also exerted therapeutic effects in myocardial ischemia/reperfusion (Lv et al., 2018; Lv et al., 2021), liver (Hong et al., 2020) and cerebral ischemia/reperfusion injuries (Liu et al., 2020; Luo J. et al., 2021). Our study investigated the neuroprotective effects and neurological improvement effects of 6-gingerol on SAH-induced EBI. Initially, we confirmed that SAH rats benefited from the brain-protective effects of 6-gingerol, consistent with previous reports (Liu et al., 2020; Luo Y. et al., 2021). Moreover, 6-gingerol effectively attenuated neuronal apoptosis post-SAH.

To gain insight into the molecular mechanism underlying the 6-gingerol-mediated neuroprotective effect on SAH-induced EBI, we performed RNA-sequencing to compare the transcriptomes of SAH + 6-gingerol rats and control rats. KEGG and GO enrichment analyses results indicated regulation of cell apoptosis and immune response to be the main mechanisms of 6-gingerol in SAH rats. These results are consistent with the main pharmacological effects of 6-gingerol on myocardial ischemia injury and hypoxic-ischemic brain injury (Lv et al., 2018; Kang et al., 2019; Luo J. et al., 2021). We looked into possible direct molecular targets of 6-gingerol from the list of the differentially expressed genes, and found that *GBP2* was one of the lowest expressed genes in the SAH + 6-gingerol treatment group. GBP2, which belongs to the dynamin superfamily of interferon-gamma-inducible large GTPases, is induced by IFN-γ and widely expressed in cells in a variety of organisms (Martínez-Lumbreras et al., 2016). Among the published reports, GBP2 has been shown to be a mediator of tumor (Yu et al., 2020) and intracellular immunity (Ma et al., 2008; Traver et al., 2011; Kotov et al., 2019). More importantly, GBP2 participates in the regulation of traumatic brain injury (Miao et al., 2017; Xiao et al., 2020). As Miao et al. reported, GBP2 protein levels significantly increased after brain injury and overexpression of GBP2 in neuronal cells aggravated neuronal apoptosis, (Miao et al., 2017). Our study found that GBP2 protein expression significantly increased following SAH and reached a peak at 24 h post-SAH, indicating that GBP2 may exert a role in the SAH process. Immunofluorescence staining results showed intense GBP2/NeuN co-localization, which was not evident with either Iba-1 or GFAP, suggesting that GBP2 might affect neurons exclusively post-SAH.

Furthermore, GBP2 expression was significantly reduced in rats of the SAH + 6-gingerol group. However, deterioration of neurologic function and enhancement of cerebral edema were observed in rats of the SAH + 6-gingerol + rGBP2 group. Additionally, compared with the SAH + 6-gingerol group, overexpression of rGBP2 significantly increased neuronal cells apoptosis. These findings indicate that GBP2, which may act as a direct target of 6-gingerol, participates in regulating SAH-induced neuronal apoptosis. KEGG and GO enrichment analyses suggested that 6-gingerol could inhibit SAH-induced inflammation; consistently, the results confirmed that 6-gingerol reduced NF-κB and p38 pathway-mediated inflammation in SAH rats (data not shown). However, GBP2 fail to co-locate with Iba-1 or GFAP, suggesting that GBP2 might not mediate the anti-inflammatory effect of 6-gingerol in SAH rats.

The PI3K/AKT pathway is recognized as a canonical pathway regulating neuronal death and survival in SAH-induced brain injury (Zhu et al., 2018; Peng et al., 2019). Numerous studies have confirmed that inactivation of the PI3K/AKT pathway is apparent in SAH rat models, and that its activation exerts protective properties against neuronal apoptosis post-SAH (Xie et al., 2018; Wu et al., 2020). Consistently, in our study, both PI3K and AKT showed a low level of phosphorylation in SAH rats, which was increased in rats of the SAH + 6-gingerol group, indicating that 6-gingerol re-activated the PI3K/AKT pathway. Moreover, cell apoptosis levels decreased following increased p-PI3K and p-AKT expression. LY294002 (PI3K inhibitor) treatment reversed the effects of 6-gingerol treatment, resulting in increased neuronal apoptosis and neurological deficits. Therefore, 6-gingerol inhibited neuronal apoptosis by activation of the PI3K/AKT pathway in SAH rats. A previous study confirmed that GBP2 induced neuronal apoptosis in traumatic brain injury, and that GBP2 inhibited the PI3K/AKT pathway to induce cell apoptosis in leukemia (Luo Y. et al., 2021). Here, we investigated the relationship between GBP2 and PI3K/AKT signaling in a SAH rat model and found that GBP2 overexpression significantly inhibited the activation the PI3K/AKT pathway in rats of the SAH + 6-gingerol group. Accordingly, cell apoptosis levels increased after GBP2 overexpression in rats of the SAH + 6-gingerol group. Finally, GBP2 expression did not change with the LY294002 treatment. Taken together, the results demonstrate that 6-gingerol activated the PI3K/AKT pathway to inhibit neuronal apoptosis via downregulation of GBP2.

There are several limitations to our study. First, 6-gingerol can exert various neuroprotective effects. However, we evaluated only the neuronal apoptosis without neuroinflammation and oxidative damage. Secondly, only a single dose of 6-gingerol was administered, therefore multiple administrations should also be considered in the future. Finally, Whether GBP2 is the direct target of 6-gingerol remains elusive, and the precise mechanisms should be explored. Therefore, our further studies will focus on these issues.

In summary, our findings demonstrate that 6-gingerol plays a neuroprotective role in SAH rats via suppression of GBP2 expression to activate the PI3K/AKT pathway. 6-gingerol may act as a novel and promising candidate drug against SAH-induced EBI. Besides, GBP2 is also a potential therapeutic target for SAH-induced EBI.

## Data Availability

The datasets presented in this study can be found in online repositories. The names of the repository/repositories and accession number(s) can be found below: https://www.ncbi.nlm.nih.gov/geo; GSE201312.

## Supplementary Material

Supplementary Figure S1 Experimental design and animal groups. SAH, subarachnoid hemorrhage; i.p., intraperitoneal; h, hour. PBS, phosphate buffer solution; i.c.v., intracerebroventricular; rGBP2, GBP2 recombinant protein; LY294002, PI3K selective inhibitor.

Supplementary Figure S2 6-gingerol alleviates neuronal apoptosis and microglia activation. **(A)** Representative images for NeuN immunofluorescence staining of brain sections from rats in the sham, SAH + PBS and SAH + 6-gingerol groups. Scale bar = 150 μm. **(B)** Representative images for GFAP immunofluorescence staining of brain sections from rats in the sham, SAH + PBS and SAH + 6-gingerol groups. Scale bar = 150 μm. **(C)** Representative images for Iba-1 immunofluorescence staining of brain sections from rats in the sham, SAH + PBS and SAH + 6-gingerol groups. Scale bar = 150 μm.

## Abbreviations

SAH, subarachnoid hemorrhage; EBI, early brain injury; GBP2, guanylate-binding protein; TUNEL, terminal deoxynucleotidyl transferase dUTP nick end labeling; KEGG, Kyoto Encyclopedia of Genes and Genomes; GO, gene ontology; PBS, phosphate buffered saline; DAPI, 4′,6-diamidino-2-phenylindole.
