# Supplementary material for: 6-Gingerol attenuates subarachnoid hemorrhage-induced early brain injury via GBP2/PI3K/AKT pathway in the rat model
Source: Front Pharmacol. 2022 Aug 25;13:882121. doi: 10.3389/fphar.2022.882121 (PMC9453877; doi:10.3389/fphar.2022.882121)
Supplement: Supplementary file 1 [file DataSheet1.pdf]

Supplementary Material

Supplementary Table S1. Modeling situation.

Groups	Mortality Rate	Excluded
**Experiment 1**		
Sham	0 (0/16)	0
SAH+PBS	15.79% (3/19)	3
SAH+6-gingerol (5mg/kg)	11.11% (2/18)	2
SAH+6-gingerol (10mg/kg)	15.79% (3/19)	3
**Experiment 2**		
SAH+PBS	25.00% (1/4)	1
SAH+6-gingerol (10mg/kg)	25.00% (1/4)	1
**Experiment 3**		
Sham	0 (0/15)	0
SAH (6, 12, 24, 48, 72h)	10.71% (3/28)	3
SAH+PBS	16.67% (2/12)	2
SAH+6-gingerol (5mg/kg)	16.67% (1/6)	1
SAH+6-gingerol (10mg/kg)	16.67% (2/12)	2
**Experiment 4**		
Sham	0 (0/16)	0
SAH+PBS	15.79% (3/19)	3
SAH+6-gingerol	11.11% (2/18)	2
SAH+6-gingerol+PBS	5.88% (1/17)	1
SAH+6-gingerol+rGBP2	15.79% (3/19)	3
**Experiment 5**		
Sham	0 (0/11)	0
SAH+PBS	21.42% (3/14)	3
SAH+6-gingerol	8.33% (1/12)	1
SAH+6-gingerol+DMSO	15.38% (2/13)	2
SAH+6-gingerol+LY294002	21.43% (3/14)	3

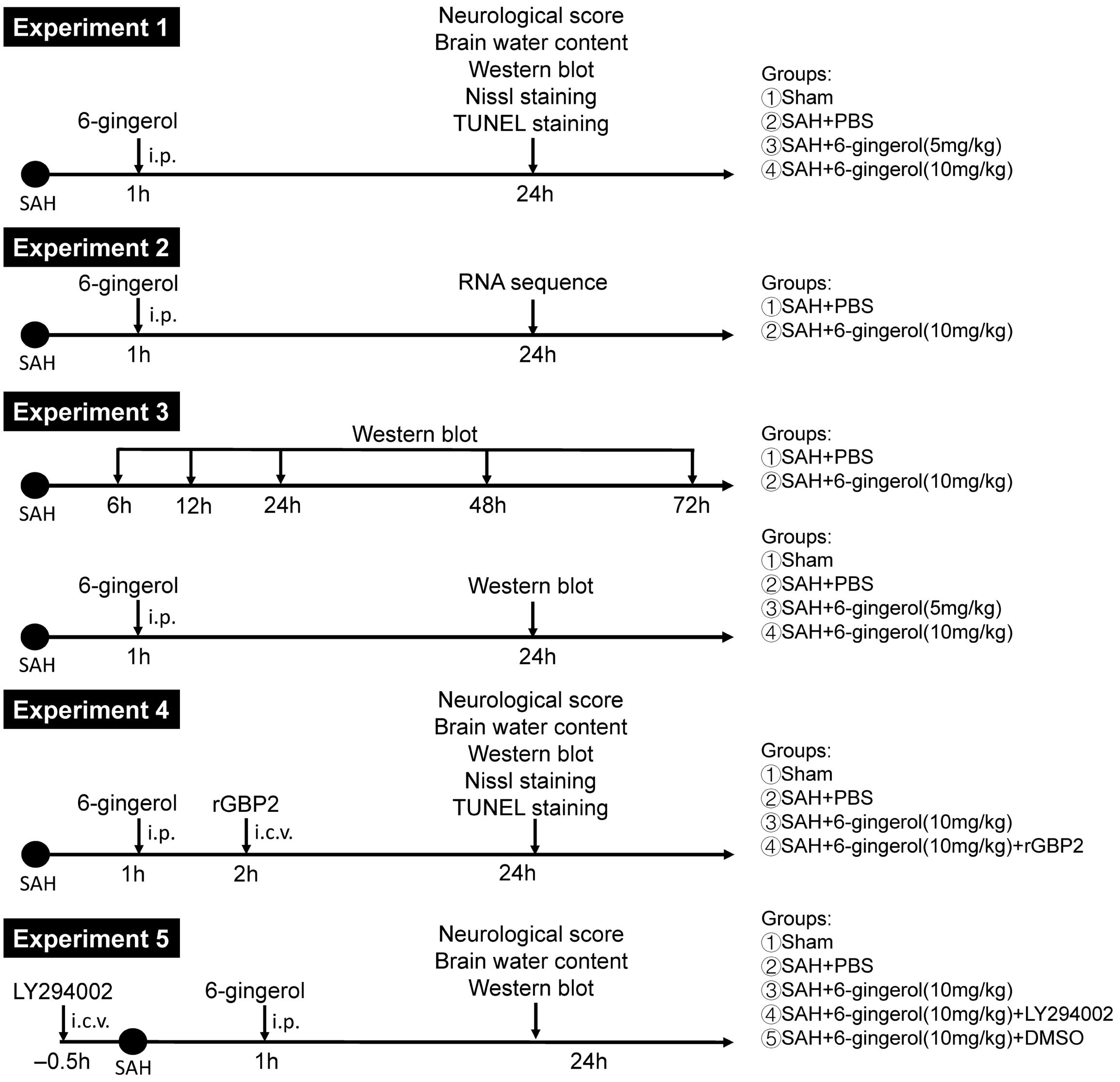

SUPPLEMENTARY FIGURE S1

Experimental design and animal groups. SAH, subarachnoid hemorrhage; i.p., intraperitoneal; h, hour. PBS, phosphate buffer solution; i.c.v., intracerebroventricular; rGBP2, GBP2 recombinant protein; LY294002, PI3K selective inhibitor

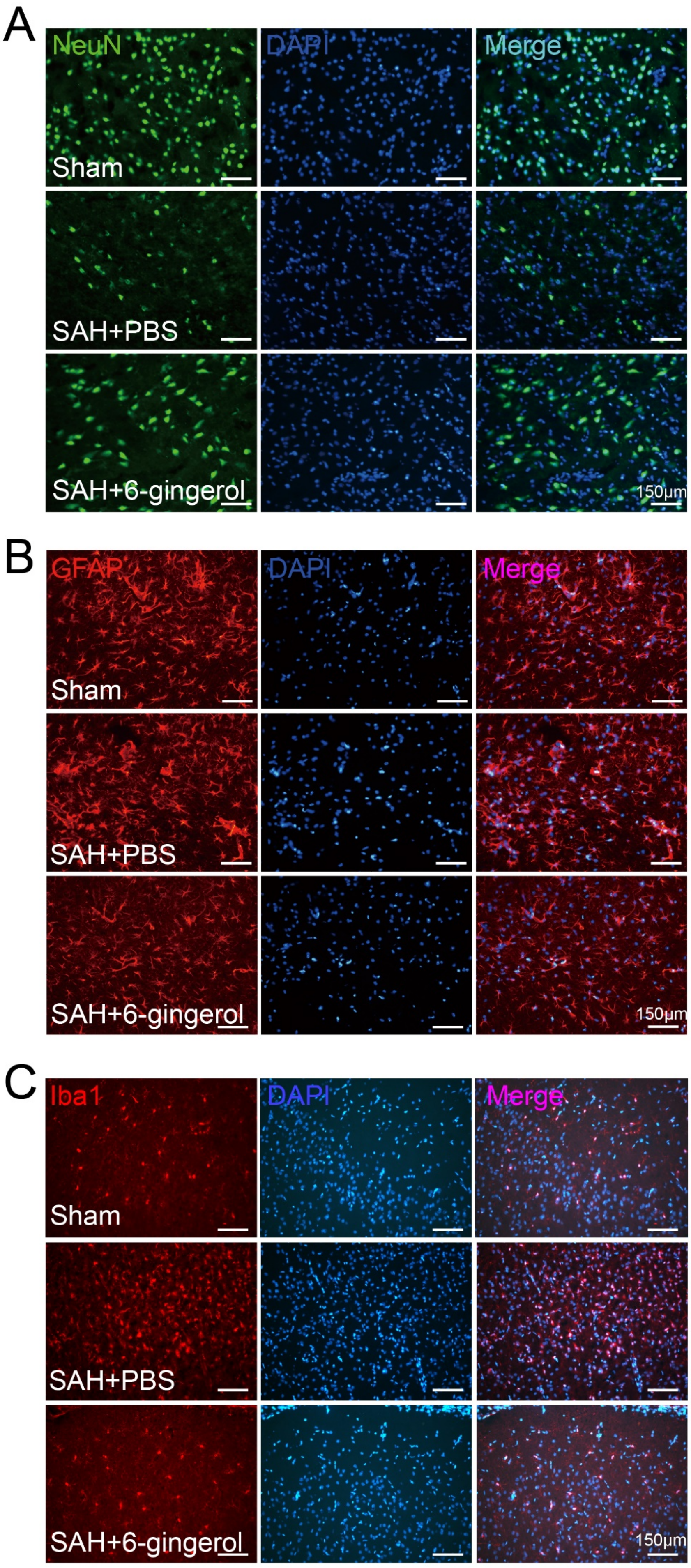

SUPPLEMENTARY FIGURE S2

6-gingerol alleviates neuronal apoptosis and microglia activation. (A) Representative images for NeuN immunofluorescence staining of brain sections from rats in the sham, SAH + PBS and SAH + 6-gingerol groups. Scale bar = 150 µm. (B) Representative images for GFAP immunofluorescence staining of brain sections from rats in the sham, SAH + PBS and SAH + 6-gingerol groups. Scale bar = 150 µm. (C) Representative images for Iba-1 immunofluorescence staining of brain sections from rats in the sham, SAH + PBS and SAH + 6-gingerol groups. Scale bar = 150 µm.
